# Could Naturally Occurring Coronaviral Diseases in Animals Serve as Models for COVID-19? A Review Focusing on the Bovine Model

**DOI:** 10.3390/pathogens9120991

**Published:** 2020-11-26

**Authors:** Jonas Johansson Wensman, Maria Stokstad

**Affiliations:** 1 Department of Clinical Sciences, Swedish University of Agricultural Sciences, SE-750 07 Uppsala, Sweden; 2 Department of Production Animal Clinical Sciences, Norwegian University of Life Sciences, 0102 Oslo, Norway; maria.stokstad@nmbu.no

**Keywords:** coronavirus, animal model, bovine, SARS-CoV-2, one health, respiratory infection, gastrointestinal infection

## Abstract

The current pandemic of COVID-19 has highlighted the importance of basic studies on coronaviruses (CoVs) in general, and severe acute respiratory syndrome CoV type 2 (SARS-CoV-2) in particular. CoVs have for long been studied in veterinary medicine, due to their impact on animal health and welfare, production, and economy. Several animal models using coronaviral disease in the natural host have been suggested. In this review, different animal models are discussed, with the main focus on bovine CoV (BCoV). BCoV is endemic in the cattle population worldwide and has been known and studied for several decades. SARS-CoV-2 and BCoV are both betacoronaviruses, where BCoV is highly similar to human coronavirus (HCoV) OC43, encompassing the same virus species (*Betacoronavirus 1*). BCoV causes respiratory and gastrointestinal disease in young and adult cattle. This review summarizes the current knowledge of the similarities and dissimilarities between BCoV and SARS-CoV-2, as well as discussing the usage of BCoV as a model for human CoVs, including SARS-CoV-2.

## 1. Introduction

Coronaviral diseases in animals have been known and studied for decades, long before the severe acute respiratory syndrome (SARS) appeared in 2002, and coronaviruses (CoVs) caught the attention of human virologists. With the current coronavirus disease 2019 (COVID-19) pandemic caused by SARS-CoV-2 (genus: *Betacoronavirus*, subgenus: *Sarbecovirus*) [1], coronaviruses have once again got attention from the scientific community. To make further progress in the basic understanding of SARS-CoV-2 and other human coronaviruses, there is a need to develop different informative model systems. Several animal models using experimental SARS-CoV-2 infection have been developed, including ferret [2], hamster [3], cat [4], and mouse, including transgenic mouse [5]. Natural infection in farmed minks [6], and occasional cases in cats [7], could also provide novel insights into SARS-CoV-2 epidemiology and pathology. One limitation of using SARS-CoV-2 infection in animal model systems is the need for BSL3 laboratory and animal facilities. The pathogenesis of an infectious disease should ideally be studied in species where the virus and host have co-evolved, and in the case of host jumps, also in other susceptible species. The animal model system also needs to be well-established in terms of virus and host genetics, and well-equipped with various analytical tools (e.g., diagnostic assays, cell culture possibilities, and assays for studying virus–host interactions). The animal model system can be studied either in experimentally infected animals, or in naturally occurring disease cases in the animal population. From an animal welfare point of view, and to meet the three R’s in animal experiments (replace, reduce, refine), studying naturally occurring coronaviral disease cases in animal populations is beneficial, and mimics the natural setting best. The advantage of experimental models is the controlled conditions. In this review, we argue that coronaviral diseases in their natural hosts could be relevant to use as models for human infections, and should be explored further, with a focus on bovine coronavirus (BCoV) infection.

## 2. Animal Coronaviral Diseases that Could Be Relevant as a Model for COVID-19

Several coronaviral diseases in animals have been suggested as models for COVID-19, mainly feline infectious peritonitis (FIP) in cats [8], canine respiratory coronavirus (CRCoV) infection in dogs [9], porcine respiratory coronavirus (PRCV) infection in pigs, and BCoV infection in cattle [8,10]. All suggested models have their pros and cons (Table 1), and the choice of model may vary depending upon what aspect of the disease is in focus. FIP is caused by one of two biotypes of feline coronavirus (FCoV), that belongs to the genus *Alphacoronavirus* [11] and consists of the two biotypes, feline enteric coronavirus (FECV), and FIP virus (FIPV). FECV causes either subclinical infection or mild diarrhea, and cats can be persistently infected. Approximately 5% of persistently infected cats develop FIP, due to mutations of the benign FECV into the fatal FIPV in the individual cat [12]. These mutations lead to a shift in the tropism of the virus, basically from epithelial cells in the gut to monocytes and macrophages. This mutation event has intrigued researchers for decades, but to the best of our knowledge, it does not reflect what is happening in COVID-19. PRCV also belongs to the genus *Alphacoronavirus* and causes mild respiratory signs in pigs [13]. This virus is a deletion mutant of the more pathogenic transmissible gastroenteritis virus (TGEV), and the spread of PRCV has provided a beneficial cross-protection in the pig population [14]. CRCoV and BCoV belong to the genus *Betacoronavirus* (subgenus *Embecovirus*), and are genetically similar to each other and to the human coronavirus (HCoV) OC43 [15]. In fact, all three viruses are classified as the same species, *Betacoronavirus 1*, and recombination events among viruses in this species occur [16]. CRCoV primarily causes upper respiratory infection [17], and is part of the canine infectious respiratory disease complex [18], commonly known as kennel cough. It is widely spread throughout the world; however, as it was rather recently detected [19], there is still largely a lack of scientific data published on CRCoV. This could be attributed to the mild clinical signs, and that although the disease can cause problems for dog owners and dog businesses, it does not cause severe societal impact. BCoV, on the other hand, is a major concern, especially in the dairy industry worldwide, causing severe economic losses due to its impact on animal welfare and health, production, and economy [20,21,22]. Hence, this virus has been widely studied and there are also several available assays for studying bovine immunity and virus–host interactions in BCoV infection. We will here, therefore, focus on BCoV as a model for COVID-19.

## 3. A Brief Summary of BCoV Experiences

BCoV is ubiquitous in cattle worldwide according to seroprevalence data. The virus causes three distinct clinical syndromes: calf diarrhea; winter dysentery (outbreak of diarrhea in adults); and respiratory infections [10,22]. The virus is shed in nasal secretions and feces. Isolates from both enteric and respiratory infections are antigenically similar, comprising a single serotype [21]. Under experimental conditions, virus from outbreaks of winter dysentery produce mainly respiratory disease in exposed calves [35]. Differences in clinical signs and severity between young and adult/old individuals are seen [36]. The reasons for these differences are still unknown and should be explored. It also remains unclear why some BCoV-infected individuals display severe signs while others remain sub-clinically infected, despite similar immune status at infection. Underlying disease or respiratory coinfections, immunosuppression, dose, and route of infection are all potential cofactors that can exacerbate the severity of BCoV infections, and enhance virus transmission or host susceptibility. Presence of antibodies in animals in a herd is not associated with less disease, in terms of lower incidence of respiratory disease or BCoV shedding in the herd [37]. Repeated nasal shedding episodes are seen in the same animal, with or without respiratory disease, but with increases in antibody titers consistent with reinfection [38]. These findings suggest a lack of long-term mucosal immunity after natural respiratory BCoV infection, although the cellular immunity has not yet been comprehensively studied. 

Prophylaxis and treatment of BCoV in the cattle population has been limited to herd-based measures to improve the animals’ robustness in general and treatment of secondary bacterial infections with antimicrobials. Development of vaccines has been challenging, and no antiviral treatment is available. In Norway, a population-based approach has been chosen to combat BCoV in cattle through improved external biosecurity [20]. The core principle is classification of all herds based on serological testing of a pooled sample from selected animals. Virus-introduction to herds is prevented through improved biosecurity measures, with no vaccination or medical treatment. Antibody positive herds are not believed to be protected against disease but could contain infectious animals. These herds are expected to become free from virus over time if a new introduction of BCoV is avoided. This approach has not only led to reduction of prevalence at the population level, but also to a unique collection of BCoV serological and clinical data that could be used for comparative studies of coronaviral diseases as discussed below. 

So far, natural infection of SARS-CoV-2 in cattle has not been reported. It has been predicted that cattle have a medium likelihood of being infected with SARS-CoV-2 [39], the same predicted grading as cats that have already been shown to be susceptible to the infection [40,41]. Experimental SARS-CoV-2 infection in cattle has however been performed, indicating that cattle are indeed susceptible to SARS-CoV-2 infection, although at a low level [42]. No cross-protection from BCoV antibodies was detected, and although the likelihood of spillover infection from human to cattle is considered to be very low [43], it should not be neglected, due to the possibility for recombination events between BCoV and SARS-CoV-2 [42]. 

## 4. Similarities and Differences—SARS-CoV-2 and BCoV

Both SARS-CoV-2 and BCoV are pneumoenteric betacoronaviruses causing upper and lower respiratory infection. Both spread easily in their host populations. Analysis of CoV genomic sequence data has revealed that BCoVs are closely related to several human CoVs [25]. Although BCoV is more related to human “common cold” CoVs, BCoV and SARS-CoV-2 can have more severe clinical outcomes and possibly a more comparable pathogenesis [10]. SARS-CoV-2 has newly emerged and spreads epidemically, while BCoV has existed for a long time, is endemic worldwide, and relatively well-known. Both vary in severity and signs, and both cause disease in a complex interplay between virus, host, and environment [21,44]. Given that both pneumonia and diarrhea occur in COVID-19 patients and BCoV infected cattle, knowledge about the pathogenesis of BCoV can contribute to a better understanding of SARS-CoV-2. 

An expert advisory group from The British Society for Immunology and Academy for Medical Sciences has published a report identifying 13 priority areas of immunological research, of high importance for tackling SARS-CoV-2 [45]. We will here follow some of these priority areas (somewhat differently grouped) and see if and how studies of BCoV infection could be useful in addressing them.

### 4.1. What is a Protective (Antibody and Cellular) Immune Response, How Long is the Duration, and How Could It Be Safely Tested in Large-Scale?

An increased understanding of the protective immunity to coronaviral diseases is urgently needed. Upon BCoV infection, an antibody response is seen within 14 days [35], and can be readily detected for years [36,46]. Reports have suggested that T-cell immunity can elicit a protective immunity to SARS-CoV-2 without a detectable antibody response [47,48]. Until now, BCoV infection has been considered to always result in an antibody response, but with unclear duration and non-complete protection. The cellular immune response of BCoV infection has, to the best of our knowledge, not yet been comprehensively studied. There is a study indicating that interleukin (IL) -2 activated lymphocytes from the intestinal epithelium, but not systemic lymphocytes, have a cytotoxic effect on BCoV-infected cells [49]; however, it is not yet known whether this effect is seen in BCoV-infected animals. Thus, cellular immunity might be more important than previously considered for protection from BCoV infection, and should be further explored. 

Revealing the duration of antibodies is key to understanding the epidemiology and herd immunity to CoV infections. Studies of duration of immunity may involve many years of longitudinal follow-up. Due to research activity for many years and ongoing control activity, we have access to serum samples and individual and herd data, providing an excellent opportunity for studying long-term immunity against CoVs. Many countries are now testing for SARS-CoV-2 antibodies in the population or in specific groups, however, no study has evaluated whether the presence of antibodies to SARS-CoV-2 confers immunity to subsequent infection by this virus in humans. The experience from cattle suggests that infected animals are seropositive for years after infection, but herds with many seropositive animals still experience severe disease outbreaks. This has led to the view that detection of antibodies is useful for detection of previous infection, but that the seropositive animals and herds are not necessarily protected from new infection. 

The structure of the cattle industry makes it natural to use the herd (farm) as the epidemiological unit in surveillance and epidemiological research. Despite considerable live animal contacts between herds (animal purchases, common pasture, etc.), this is a more confined unit than “family”, “school class”, or similar, in the human counterpart. Diagnostics in control efforts are commonly based on “herd” as the classification unit, and the sampling design for classification is based on epidemiological calculations of sensitivity and specificity of the classification at herd level. A relatively few animals can then be selected for sampling, and the material pooled before laboratory analysis (serology) is performed, where a negative result rules out the presence of virus, while a positive sample indicates previous or ongoing virus circulation. This creates a system where analysis of one sample forms the basis for classification of a herd, that may comprise of several hundred animals, which is practical and inexpensive. This is a common system for surveillance in animal populations, in contrast to the more common strategy in the human population where surveillance is generally based on identification and diagnostic confirmation of clinical suspicious cases, and usually with the individual as a unit. This different way of thinking in surveillance and diagnostics in veterinary medicine can obviously not be transferred to humans but might inspire thinking differently with regard to classifying groups or areas as free or possibly infected, for example as has been suggested in the use of wastewater for COVID-19 surveillance [50].

### 4.2. What is the Early Immune Response and Pathogenesis, and What are the Biomarkers for Severe Disease?

An understanding of the coronavirus pathogenesis and immunity in the early infection stage is urgently needed for development of measures for prevention, diagnosis, and therapy of COVID-19. Discovery of biomarkers will be of great value for treatment and prognosis of SARS-CoV-2 infection, and in particular for the early identification of individuals that will develop severe disease. Studies in model systems, such as BCoV infection in cells and in cattle, could here provide an important tool for exploring the early immune response, and in identifying biomarkers of prognostic value for COVID-19. Although BCoV infection has been studied for decades, there are knowledge gaps in the molecular pathogenesis and early immune response. In vitro studies have suggested that BCoV attaches to the host cell by sialic acids, and possibly uses the bovine leukocyte antigen class I for entry [51], whereas for SARS-CoV-2, the cellular receptor of the virus is the angiotensin-converting enzyme 2 (ACE2) [52]. One of few studies on the early immune response of BCoV infection shows that type II interferon (IFN gamma), as well as the pro-inflammatory genes IL-10 and -6 are downregulated in the intestinal mucosa [53], which could indicate a host immune evasion mechanism of the virus. Whether this is also the case for SARS-CoV-2 remains to be answered. We argue that in-depth studies on the early immune response and pathogenesis of BCoV infection would increase our understanding of coronaviral diseases in general, including COVID-19. Identifying and manipulating central biomarkers of the disease may be pivotal for the control of excessive inflammation, and a dysregulated immune response.

### 4.3. What is the Rate of Asymptomatic Spread and How Do We Know When Infectious Virus is No Longer Shed in Convalescents?

**Figure 1:**
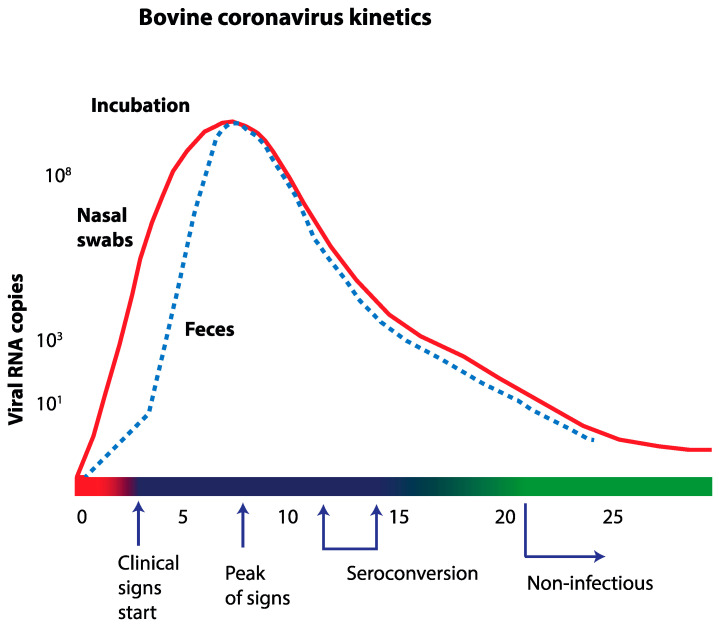
Schematic kinetics of bovine coronavirus infection based on experiences from an experimental infection in calves (see Oma et al., [35]).

Most experimental BCoV infection studies performed have used virus inoculation as the challenge procedure. In an experimental infection of calves using exposure to shedding calves as the mode of infection, the duration and quantity of BCoV shedding in nasal and fecal samples was studied [35]. Exposed calves were BCoV RNA positive by quantitative RT-PCR on day 1–12 post-exposure (Figure 1), and occasionally positive until day 28. Fecal samples were negative the first day after exposure, but day 2–17 at least half of the exposed group shed virus in feces, and then BCoV RNA was intermittently detected until day 35. This intermittent shedding had already started at day 14, and the fecal samples from calves could be negative for up to six days, before again being positive. In general, BCoV RNA detection and clinical scoring of general depression and cough had a good correlation. Interestingly, all exposed calves shed viral RNA at least one day before showing clinical signs. Thus, pre-symptomatic spread could occur. The peak of viral shedding occurred more than a week later than the fever peak [35]. All exposed calves seroconverted by day 14, and after that the viral shedding dropped. In this study, two sentinel calves were allowed to commingle with the exposed group three weeks after the initial exposure. Although some of the exposed calves still had detectable levels of BCoV RNA in nasal secretions and feces, the sentinel calves remained uninfected. The authors hypothesized that the PCR positivity reflected antigen–antibody complexes, where the virus was neutralized and no longer infectious. 

It is difficult to directly compare this study with similar studies in SARS-CoV-2 infection, especially as many mild COVID-19 cases do not seem to lead to seroconversion. Studies are also indicating a variation in viral RNA shedding from 1–35 days, likely depending on study design [54]. In one study, the median duration of viral RNA shedding was 14 days, and no factors affecting the duration of shedding were identified [55]. Similarly to the BCoV study [35], intermittent shedding was found, and then the total duration of shedding was longer [55]. The authors also found an association between prolonged SARS-CoV-2 RNA shedding and lower levels of cytokines involved in T-cell activation [55]. Thus, even though seroconversion is not always seen after SARS-CoV-2 infection, immune status is important in regulating the duration of viral shedding. It is also important to remember that viral RNA shedding is not the same as shedding of infectious virus [35,54]. Further studies on how the humoral and cellular immunity affects shedding of infectious virus particles and the duration of infectivity are thus needed, and controlled experiments using BCoV infection could also be helpful.

An important issue in line with this is also the survival of infectious virus particles on fomites and the indirect transmission routes. For BCoV, high viral RNA loads, and in some cases, infective virus particles were found on equipment up to 24 h after being exposed to BCoV-infected calves, although the equipment was cleaned, but not disinfected [56]. Even though the persistence of SARS-CoV-2 on various materials is low [57], the risk for indirect transmission via fomites should not be neglected, especially in conditions where high levels of viral shedding can be suspected. There is a current debate about whether SARS-CoV-2 can be transmitted by aerosols or only by droplets [58]. For BCoV infection, direct contact and indirect transmission by fomites are the most likely route of transmission between herds. However, there were indications of airborne transmission during an experimental infection, as even control calves got infected by BCoV, despite all biosecurity precautions being taken and the calf groups being separated 30 m apart [59].

### 4.4. What is the Role of Immunogenetics?

There are indications of genetic factors having a role in severity of COVID-19 [60]. In this case, a genetic variant of the toll-like receptor (TLR) 7 gene, leading to impaired type I and II IFN response upon SARS-CoV-2 infection. To the best of our knowledge, no studies of genetic factors in BCoV infection have been published so far. Such studies would, however, be possible to initiate, as BCoV infection is frequently occurring, many dairy cows are already genotyped as part of breeding programs, and powerful tools for performing a genome-wide association study (GWAS) are at hand. Thus, here natural BCoV infection in dairy herds could provide candidate genes, interesting to study further in human coronaviral diseases, including COVID-19.

## 5. From Humans to Cattle and Back to Humans

Several of the current knowledge gaps for COVID-19 are crucial, but challenging, to study in humans. Methodologies and research material from veterinary medicine, such as natural and experimental infection, euthanasia, and long-term testing of randomly selected animals with controlled exposure would, in our opinion, be highly beneficial as a model for COVID-19, and provide a novel understanding on coronaviral diseases in general. Experimental infection in cattle is expensive, labor-demanding, and usually requires BSL-2 facilities for large animals, resulting in a low number of animals being included. On the other hand, natural disease outbreaks in cattle in the field provide large number of observations for studies on CoV biology, including sequential euthanasia and pathological examinations, that would be useful to clarify, for example, the proposed occurrence of viral persistence [35,61,62]. Easy access to organ material at abattoirs is another advantage for establishing ex vivo models for exploring early mucosal immune responses during BCoV infection, not possible, or extremely challenging, to perform in human medicine. Based on the created knowledge from natural disease outbreaks and ex vivo models, carefully planned experimental BCoV infections, even in a few animals, can deliver important validation of the results. Identification of candidate biomarkers and immune functions from cell and animal experiments need to be thoroughly validated on material from humans, in order to ensure that the findings are of true relevance for COVID-19. To our knowledge, no research groups are using BCoV as a model for studying the urgent knowledge needs for COVID-19. Cattle could provide a unique model for comparative immunological research, as most immunological signaling systems are evolutionary conserved in mammals [63], and the majority of immune cell phenotypes and functional characteristics show distinct similarities with humans [63,64].

We argue that joint efforts from human and veterinary medical scientists would be useful to develop the understanding of CoV biology, where the research questions are generated from human medicine, research methodology, and biomaterial from veterinary science, and validation of the results is done in humans. 

## Figures and Tables

**Table 1:** A comparison of various animal coronaviruses that have been proposed as models for SARS-CoV-2 infection.

	Feline Infectious Peritonitis Virus	Porcine Respiratory Coronavirus	Canine Respiratory Coronavirus	Bovine Coronavirus	Severe Acute Respiratory Syndrome Coronavirus Type 2
Genus	*Alphacoronavirus*	*Alphacoronavirus*	*Betacoronavirus*	*Betacoronavirus*	*Betacoronavirus*
How common?	Worldwide, but only sporadic cases [11]	Worldwide [14]	Worldwide, and probably very common [18]	Endemic worldwide [21,22]	Pandemic
Disease characteristics	Highly variable clinical signs incl. pleural and abdominal effusions, fever, lethargy, anorexia, and weight loss; fatal [11]	Respiratory signs, incl. pneumonia, but most often mild to moderate clinical signs [13]	Upper respiratory signs (part of the canine infectious respiratory disease complex); often mild [17]	Diarrhea and respiratory signs; part of bovine respiratory disease complex and winter dysentery in adult cows can lead to severe signs [10,22]	Upper respiratory signs, ranging from mild to severe (incl. acute respiratory distress); in severe cases multi-organ failure; gastrointestinal signs; occasionally, long-term effects [23,24]
Genetic characteristics	Arises from mutations of the benign feline enteric coronavirus in the individual cat [12]	Deletion mutant of the more pathogenic transmissible gastroenteritis virus (TGEV) [14]	Genetically similar to bovine coronavirus (BCoV) and human coronavirus (HCoV) OC43 [15]	Genetically similar to canine respiratory coronavirus (CRCoV) and HCoV OC43 [15,25]	Genetically similar to bat SARS-like CoVs [26]
Tropism	Multiple, mainly monocytes/macrophages [11]	Respiratory tract (epithelial cells and alveolar macrophages) [13,14]	Mainly respiratory tract [17]	Respiratory and gastrointestinal tract [10]	Respiratory and gastrointestinal tract (incl. epithelial cells and alveolar macrophages) [23,24,27,28]
Vaccine?	Yes, but only useful in a seronegative population due to antibody dependent enhancement [29,30]	No [30]	No [30]	Yes, but mainly for boosting the colostrum and for enteritis in calves [30]	Several under development [31]
Treatment?	Nucleoside analog GS-441524, similar to remdesivir [32]	No	No	No	Remdesivir, depending on severity and stage of disease [33]; several other treatments also undergoing clinical trials [34]
